# Design and implementation of a prototype radiotherapy menu in a patient portal

**DOI:** 10.1002/acm2.14201

**Published:** 2023-11-09

**Authors:** Kayla O'Sullivan‐Steben, Luc Galarneau, Susie Judd, Andrea M. Laizner, Tristan Williams, John Kildea

**Affiliations:** ^1^ Medical Physics Unit McGill University Montreal Quebec Canada; ^2^ Research Institute of the McGill University Health Centre Montreal Quebec Canada; ^3^ Ingram School of Nursing McGill University Montreal Quebec Canada; ^4^ Gerald Bronfman Department of Oncology McGill University Montreal Quebec Canada

**Keywords:** patient education, patient portal, radiotherapy, stakeholder co‐design

## Abstract

**Purpose:**

Radiotherapy patients often face undue anxiety due to misconceptions about radiation and their inability to visualize their upcoming treatments. Access to their personal treatment plans is one way in which pre‐treatment anxiety may be reduced. But radiotherapy data are quite complex, requiring specialized software for display and necessitating personalized explanations for patients to understand them. Therefore, our goal was to design and implement a novel radiotherapy menu in a patient portal to improve patient access to and understanding of their radiotherapy treatment plans.

**Methods:**

A prototype radiotherapy menu was developed in our institution's patient portal following a participatory stakeholder co‐design methodology. Customizable page templates were designed to render key radiotherapy data in the portal's patient‐facing mobile phone app. DICOM‐RT data were used to provide patients with relevant treatment parameters and generate pre‐treatment 3D visualizations of planned treatment beams, while the mCODE data standard was used to provide post‐treatment summaries of the delivered treatments. A focus group was conducted to gather initial patient feedback on the menu.

**Results:**

Pre‐treatment: the radiotherapy menu provides patients with a personalized treatment plan overview, including a personalized explanation of their treatment, along with an interactive 3D rendering of their body, and treatment beams for visualization. Post‐treatment: a summary of the delivered radiotherapy is provided, allowing patients to retain a concise personal record of their treatment that can easily be shared with future healthcare providers. Focus group feedback was overwhelmingly positive. Patients highlighted how the intuitive presentation of their complex radiotherapy data would better prepare them for their radiation treatments.

**Conclusions:**

We successfully designed and implemented a prototype radiotherapy menu in our institution's patient portal that improves patient access to and understanding of their radiotherapy data. We used the mCODE data standard to generate post‐treatment summaries in a way that is easily shareable and interoperable.

## 1 INTRODUCTION

In this era of burgeoning Big Data and AI research in radiation oncology, researchers and clinicians increasingly seek large amounts of real‐world radiotherapy data. While these data exist, they are difficult to access due to legal protections, isolated databases, and interoperability issues.^1^, ^2^ Patients also seek access to their radiotherapy data, as knowledge about one's disease and treatment is empowering and leads to reduced anxiety and potentially better treatment outcomes.^3^, ^4^, ^5^, ^6^, ^7^, ^8^ But, radiotherapy data are typically only viewable using specialized treatment planning software applications, and their complexity makes it difficult for most patients to understand them without additional context. Therefore, our goal was to design and implement a prototype system to allow patients access to their radiotherapy data via our patient portal such that they are educational and easily shareable between patients, clinicians, and researchers.

### 1.1 Background

Radiotherapy is a cancer treatment modality in which targeted ionizing radiation is delivered to a tumor to destroy cancerous cells. It is a frequently used modality, with over 50% of cancer patients receiving radiotherapy over the course of their disease.^9^ Despite its prevalence, radiotherapy is poorly understood by the general public^7^, ^10^ and most patients experience significant anxiety prior to treatment. This anxiety may be due to misconceptions about the treatment, fear about radiation and its potential side effects, as well as an inability to visualize the treatment process.^6^, ^7^, ^11^, ^12^ Importantly, it can result in decreased quality of life, lower compliance with clinician advice and sometimes refusal to undergo treatment.^6^, ^11^, ^13^, ^14^ It is, therefore, imperative that patients are appropriately informed about their radiation treatment in a way that demystifies the process and addresses any concerns they may have about it.

As it stands, conventional radiotherapy education may fail to meet patient information needs.^6^, ^11^, ^14^, ^15^, ^16^, ^17^ Typically, radiotherapy patients are given high‐level information about their treatments verbally during appointments, and when more detailed written information is provided, it is usually not personalized to the patient's own situation. There are several limitations with the conventional approach. First, many patients find it difficult to retain all the information provided to them verbally during appointments.^11^, ^18^ Second, verbal and written communication are insufficient to demystify the more intimidating aspects of radiotherapy, such as the machine motion and the delivery of the radiation itself.^10^, ^19^ Finally, the patient is not informed regarding their own specific treatment plan. This last point is particularly important because studies have shown that educational material that is tailored to a patient's individual treatment plan can significantly increase satisfaction and reduce anxiety.^4^, ^20^, ^21^, ^22^

### 1.2 Radiotherapy patient education

Various solutions have been proposed and examined to improve patient education in radiotherapy. For instance, educational videos have been shown to be effective tools for increasing knowledge and reducing patient anxiety compared to traditional pamphlet‐based information.^23^, ^24^ Williams et al.^25^ further demonstrated the benefit of using videos containing real footage augmented with computer‐generated 3D visualizations to describe the radiotherapy treatment process and rationale. Although effective and innovative, these video‐based education approaches lack the personalization that could be provided by using a patient's own treatment plan.

A more personalized approach was proposed in a study by Atwood et al.,^26^ where radiotherapy patients were offered medical physics consultations during which they were shown personalized infographics of their own radiotherapy plans prior to treatment. Although these consultations were demonstrated to reduce patient anxiety, they required substantial departmental resources in order to train and liberate medical physicists for the purpose, which might hinder sustainability and deter their more widespread adoption in clinical practice.

Although not specific to radiotherapy, another strategy that has been shown to reduce patient anxiety surrounding their care is providing patients with direct access to their personal health records. Numerous studies have demonstrated that patients are more empowered and better informed about their care when they have access to their own healthcare data.^3^, ^5^, ^27^, ^28^ Providing such access allows patients to review and digest the information on their own time and prepare informed questions for their next consultations. In particular, patient portals—secure extensions of healthcare institutions’ medical records—are increasingly important tools that allow patients to access their healthcare data. Most patient portals contain all or some of the following features: laboratory test results, appointment schedules, and prescription medication history.^29^, ^30^, ^31^, ^32^, ^33^ While the use of patient portals has shown great promise in improving patient education, some healthcare data still remain stubbornly inaccessible to patients. Barriers preventing such access include security concerns, interoperability issues, and uneasiness about patients being unable to interpret their data.^1^, ^2^, ^3^, ^33^

Radiotherapy treatment plans are among the healthcare data that are not readily accessible to patients. This is in part because these data are typically only viewable using specialized treatment planning software applications that are capable of interpreting and displaying data communicated in the DICOM‐RT format.^34^ Moreover, the complexity of radiotherapy data makes them difficult for most patients to understand without additional context or access to technical expertise.

### 1.3 Our approach

We sought a solution to surmount the aforementioned issues and provide patients with access to their radiotherapy treatment plans via our in‐house‐developed patient portal, known as Opal,^35^ in a way that is both educational to the patient and easily shareable by the patient with their wider care team. In this article, we describe our approach to the design and development of a prototype radiotherapy menu in Opal as well as initial patient feedback on it. To overcome interoperability issues that typically hinder the exchange of medical data, we employed modern radiotherapy data communication standards, namely, DICOM‐RT^34^ and mCODE (minimal Common Oncology Data Elements).^36^ mCODE is a recently‐published data standard based on HL7 FHIR (Fast Healthcare Interoperability Resources) that is being developed by a large community of oncology stakeholders.^36^ To our knowledge, our work is the first prototype of a patient portal that offers patients access to their personal radiotherapy treatment plans and the first use of mCODE by a patient portal.

## 2 METHODS

### 2.1 Setting

Our comprehensive cancer center incorporates a radiotherapy clinic that houses seven linear accelerators and treats approximately 2500 patients with radiation per year. Patient data and treatment plans are managed using the ARIA oncology information system (Varian Medical Systems, Inc. Palo Alto, California, USA). Currently, the printed educational materials provided to patients are mostly not personalized. Rather, patients are given a booklet (on paper and/or in Opal) explaining the general radiotherapy process, and an additional pamphlet with one to a few pages of disease‐specific information.

**FIGURE 1:**
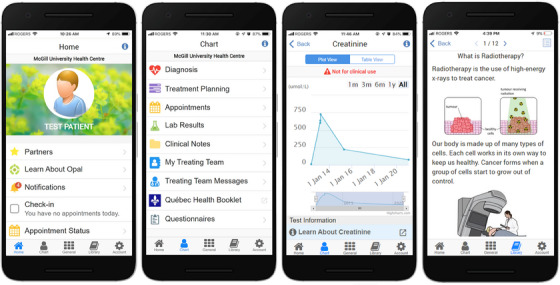
Screenshots of the Opal patient portal app at the outset of our project. The rightmost screenshot shows the type of non‐personalized radiotherapy information that was provided to patients at the outset of the project.

Patients at our cancer center have the option of downloading and registering for the Opal patient portal (Figure 1, opalmedapps.com), which is available as a smartphone application. Since its release in 2018, Opal has over 5000 users and has overwhelmingly received positive feedback.^8^, ^35^ At the outset of our project, Opal provided patients with access to certain healthcare data, such as appointments, radiation oncology clinical notes (physician‐written consultation notes, end‐of‐treatment notes, and follow‐up notes), and lab results, but not radiotherapy treatment plans. It also provided patients with educational materials (electronic booklets, videos, pamphlets) personalized to their diagnoses and treatment stages (pre‐treatment, during treatment, post‐treatment), but not specific to their treatment plans.

### 2.2 Needs assessment

**FIGURE 2:**
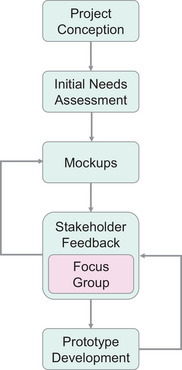
Overview of our project's workflow and its iterative stakeholder co‐design process.

An overview of our project workflow is presented in Figure 2. Before beginning software design and development, we performed an initial needs assessment regarding radiotherapy patient education broadly and at our center specifically, incorporating a literature review and informally gathered input from our patient and clinician partners. We followed the same design approach that our team had taken when designing Opal in the first place, namely, iterative participatory stakeholder co‐design.^35^ This ensured that all stakeholders were involved in providing ongoing feedback throughout the project. Because the Opal development team already had weekly one‐hour co‐design meetings established with radiotherapy patients, clinicians, software developers, and researchers, we piggybacked on these existing meetings to obtain the stakeholder feedback we needed to advance our project.

To achieve both patient and clinician buy‐in, we set two general requirements for our project at the outset. Namely, our solution should (a) not increase the already‐heavy workload of healthcare workers, and (b) be intuitive and easily understandable to patients.

To meet requirement (a), it was imperative that Opal should interface with our oncology information system with minimal effort required of the clinical team. Furthermore, we identified that it would be important that our final product be sufficiently flexible and scalable to be able to integrate with the oncology information systems of different centers to allow future widespread adoption. Thus, we determined that the radiotherapy data used in this project should be exchanged using current radiotherapy data standards.

To meet requirement (b), we needed to find a way to ensure that the radiotherapy data sent to the patient are presented in a way that meets the patient's information needs while being intuitive and easily understood. In particular, patients have reported a desire to visualize what their treatments will look like, to know what their positionings will be, and to understand some of the technical aspects of their treatments.^11^, ^17^ A previously‐reported survey conducted among our cancer patient population^35^ found that 71.7% of respondents (*n* = 233) would like to view their personal radiotherapy treatments (consisting of beam configurations and areas of the skin that might be affected by radiation) in a patient portal. We found that this number increased to 94% in a question to Opal users who are radiotherapy patients that was administered as part of an Opal quality improvement questionnaire distributed in Opal itself (see Supplemental Materials). Therefore, we concluded that the most appropriate solution should contain both textual and graphical descriptions of the treatment plan.

Finally, the timing of information provision was also considered. Literature suggests that radiotherapy patients are most anxious and prefer to receive information before the start of treatment,^11^, ^17^, ^37^ thus it would be most beneficial if patients are shown their planned radiotherapy ahead of the first treatment fraction. However, clinician stakeholders emphasized that modifications often occur over the course of treatment such that the final delivered course of radiotherapy may differ from the initial plan. As such, from a record‐keeping perspective, it could be misleading to provide patients with only their pretreatment data. A solution with good clinician acceptability, therefore, needed to additionally reflect the patient's delivered treatment record once their treatment course is complete.

A summary of the findings of our needs assessment is presented in Table 1.

**TABLE 1:** Summary of the needs assessment for our patient portal's novel radiotherapy menu.

Need	Fulfillment of need
(1) Encourage ongoing participation of stakeholders to ensure that the final product meets stakeholder needs.	Use participatory stakeholder co‐design.
(2) Efficient, portable, and secure data transfer to the patient.	Leverage the Opal patient portal's existing secure data communication infrastructure.
(3) Compatibility with existing and future healthcare systems.	Use international data standards (DICOM‐RT and mCODE).
(4) Complex data should be easily understood by patients.	Contextualize all data with textual descriptions and realistic visuals.
(5) Account for treatments that are not delivered as planned.	Distinguish between pre‐ and post‐treatment plans and ensure that the final treatment record is correct.

### 2.3 Software development code base

As the Opal patient portal was developed by our research team and was thus available to us as source code, we used it as the base software on which to build our personalized radiotherapy patient education solution. Rather than developing a new standalone software application to transfer just radiotherapy data to patients, we chose to leverage Opal's existing infrastructure, as it was already integrated with our hospital's information systems and was built with a modular design pattern that facilitates the addition of new features.^35^ This strategy not only addressed our technology needs and got us off the ground running, it also helped to reduce the learning curve that patients would otherwise encounter if faced with an additional patient portal application.

To overcome interoperability issues that typically hinder data exchange, we chose to employ radiotherapy data communication standards when transferring patient data to the patient portal application, namely, the DICOM‐RT^34^ and mCODE standards.^36^

### 2.4 Software development and evaluation

Our software additions to Opal were developed by a graduate student in medical physics whose background is in physics and computer science. The student's background was highly suited to the work and provided them with an appropriate mix of radiotherapy knowledge and an ability to quickly learn the necessary software development skills. The student was embedded in the Opal software development team, supervised by the Opal development team lead, attended daily huddles, and participated in sprints. Development was conducted following Opal's development standards and the Agile development framework.^38^ The development phase lasted approximately 6 months. Iterative design was achieved using the stakeholder feedback provided in the weekly meetings with patient partners, clinicians, software developers, and researchers. Patients and clinicians provided important feedback on mockups and incremental software development. The software developers and researchers ensured technical feasibility and adherence to standards.

In collaboration with our stakeholders, we conceptualized three main interfaces necessary to address patient needs under a single new section in the Opal app, named the radiotherapy menu:

1. A descriptive interface—provide a text and image‐based explanation of the patient's own treatment plan.
2. An interactive data visualization interface—provide an interactive 3D visual summary of the patient's treatment plan, including the patient's body contour and planned treatment beams.
3. Post‐treatment summary interface—provide a shareable and standardized end‐of‐treatment summary of the patient's actually delivered plan.

Over the course of the project, stakeholders iteratively provided feedback on the design, and modifications were made accordingly. Once the final prototype was finished and approved by our patient partners, we sought additional high‐level feedback via a focus group involving patients who had not previously been exposed to the concept of the project. Again, we piggybacked on an existing framework set up by the Opal development team. Patients were recruited via invitation notifications sent through Opal to participate in a focus group on the use of Opal for research and data sharing, including sharing of radiotherapy data. Out of the 832 notifications sent, 42 patients expressed interest in potentially participating in the focus group, and eight patients ultimately returned signed consent forms. Due to scheduling conflicts, the final focus group had four participants.

## 3 RESULTS

### 3.1 Software architecture

**FIGURE 3:**
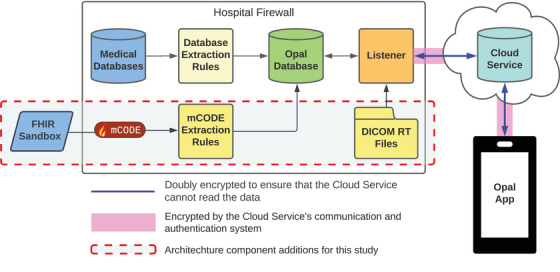
Overview of Opal's communication architecture. Certain data are copied from the hospital's Medical Databases to the Opal Database using Database Extraction Rules that determine the timing and content of the data transferred. Upon authenticated request, the Listener sends encrypted data from the Opal Database to the Opal App through a relay Cloud Service that allows for the secure communication of encrypted data from within the hospital firewall to a patient's phone.

Our software additions to Opal were built following the Opal patient portal's existing modular architecture. Opal's communication architecture (Figure 3) allows for the secure transfer of data from a hospital's databases to a patient's smartphone. Inside the hospital, select data are extracted from the Medical Databases using Database Extraction Rules and aggregated for easy access into the Opal Database. When a request for data is made from the Opal App, the Listener queries the requested data from the Opal Database and sends them from within the hospital firewall to the patient‐facing app via a secure real‐time relay Cloud Service. The Opal App receives the data and displays them to the patient. Although our software additions followed the same architectural design as the existing patient portal system, since we were building a prototype, we used synthetic patient data rather than extracting data directly from the Medical Databases.

### 3.2 Software design

**FIGURE 4:**
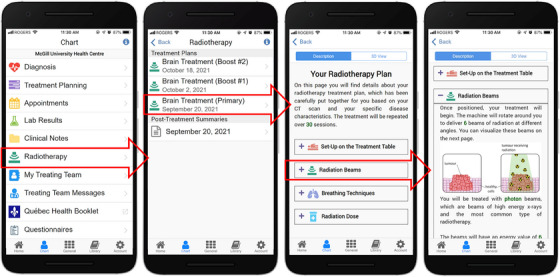
Screenshots of our prototype radiotherapy menu. The radiotherapy menu can be accessed via the Chart section in Opal, as seen in the leftmost screenshot. In the three rightmost screenshots, the main radiotherapy menu is shown along with the pre‐treatment explanatory text interface. Outlined red arrows show the interactive menu items that the user may tap on to open up the screenshots to their right. The DICOM‐RT files used to generate this plan were retrieved from the publicly available datasets in the SlicerRtData GitHub repository.^40^ ^.^

As shown in Figure 4 (left), our prototype radiotherapy menu is divided into two sections: (1) *Pre‐Treatment Plans*, consisting of descriptive text and 3D visual interfaces of the plan to be delivered, and (2) *Post‐Treatment Summaries*, which presents a concise record of the actually delivered treatment(s).

#### 3.2.1 Pre‐treatment plans

Pre‐treatment summaries are generated using data encoded in the DICOM‐RT standard, specifically, the Structure Set and Plan objects.^34^ To minimize the amount of storage space these data consume in the Opal Database, the directory path to the patient's relevant DICOM files (on a secure internally hosted network server) is stored in the database rather than the files themselves. To extract data from these files using the Listener application (Figure 3, inside the dashed red box), we use the NodeJS‐based *dicomParser* library^39^ (version 1.8.7) to parse the DICOM byte streams. Relevant parameters are extracted from the parsed information, encrypted, and sent to the Opal App to be decrypted and rendered into two pre‐treatment explanatory interfaces, one for text and one for 3D visualizations, as described below.

##### Pre‐treatment explanatory text interface

To create the pre‐treatment explanatory text interface, it was important to initially determine which aspects of their treatment plans patients wished to receive more information about. Following consultations with our patient and clinician partners, we composed educational material samples describing patient positioning on the treatment table, radiation beams, breathing techniques, and radiation dose. Once these texts were approved by our patient and clinician partners, they were coded into the Opal App as fill‐in‐the‐blank style templates. To personalize these templates for an individual patient, relevant treatment parameters are first extracted from the patient's DICOM‐RT Plan file in the Listener application (number of fractions, patient position, number of beams, beam type, beam energies, target dose). These parameters are subsequently sent to the Opal App, and the templates are dynamically filled in with different explanations according to their values.

Figure 4 presents screenshots of the prototype pre‐treatment explanatory text interface. Left to right, the screenshots show how the patient can navigate to the radiotherapy menu, open it to see their list of treatment plans and navigate through the textual description of each plan.

##### Pre‐treatment explanatory 3D visualization interface

To create the pre‐treatment explanatory 3D visualization interface, 3D models of both the patient's body and the planned treatment beams need to be constructed.

First, we generate a 3D rendering of the patient's body using the body contour that was previously drawn on the patient's CT scan during the radiotherapy planning stage. This contour information is stored as a set of 3D points for each image slice. For each patient, their body contour data are extracted on demand from their DICOM‐RT Structure Set file by the Listener application. Still in the Listener, each slice is fitted with a spline curve, from which a fixed number of equally distanced points are sampled to reduce the data transfer size to the Opal App. These sampled points are then sent to the Opal App and processed by it using a custom‐written lightweight JavaScript algorithm that efficiently triangulates the points to reconstruct a 3D shell of the patient's body.

Next, to construct the 3D beams, radiation beam information is extracted on demand from the patient's DICOM‐RT Plan file by the Listener application (gantry angle, collimator angle, jaw positions, source‐to‐axis distance, and isocenter). From these data, the point source and the four corners of the beam field at isocenter are calculated in the patient coordinate system in the Listener and then triangulated to form a 3D beam structure in the Opal App. This process is repeated for each beam.

Finally, *Three.js* ^41^ (version 0.124.0), an open‐source JavaScript library for creating animated 3D graphics, is employed in the Opal App to generate 3D meshes from the triangulated body and beam data and to subsequently render these objects on screen. Three.js’ built‐in zoom, rotation and panning features were also enabled, allowing patients to fully interact with their 3D model using smartphone touch gestures. Additional features were added into the display to enhance patients’ interaction with the treatment plan. For instance, checkboxes corresponding to each beam can be toggled to view beams individually or in combination. Additionally, the portions of the skin within the beams’ paths are colored to indicate where the skin may be affected by radiation, which can serve as a visual guide for patients.

**FIGURE 5:**
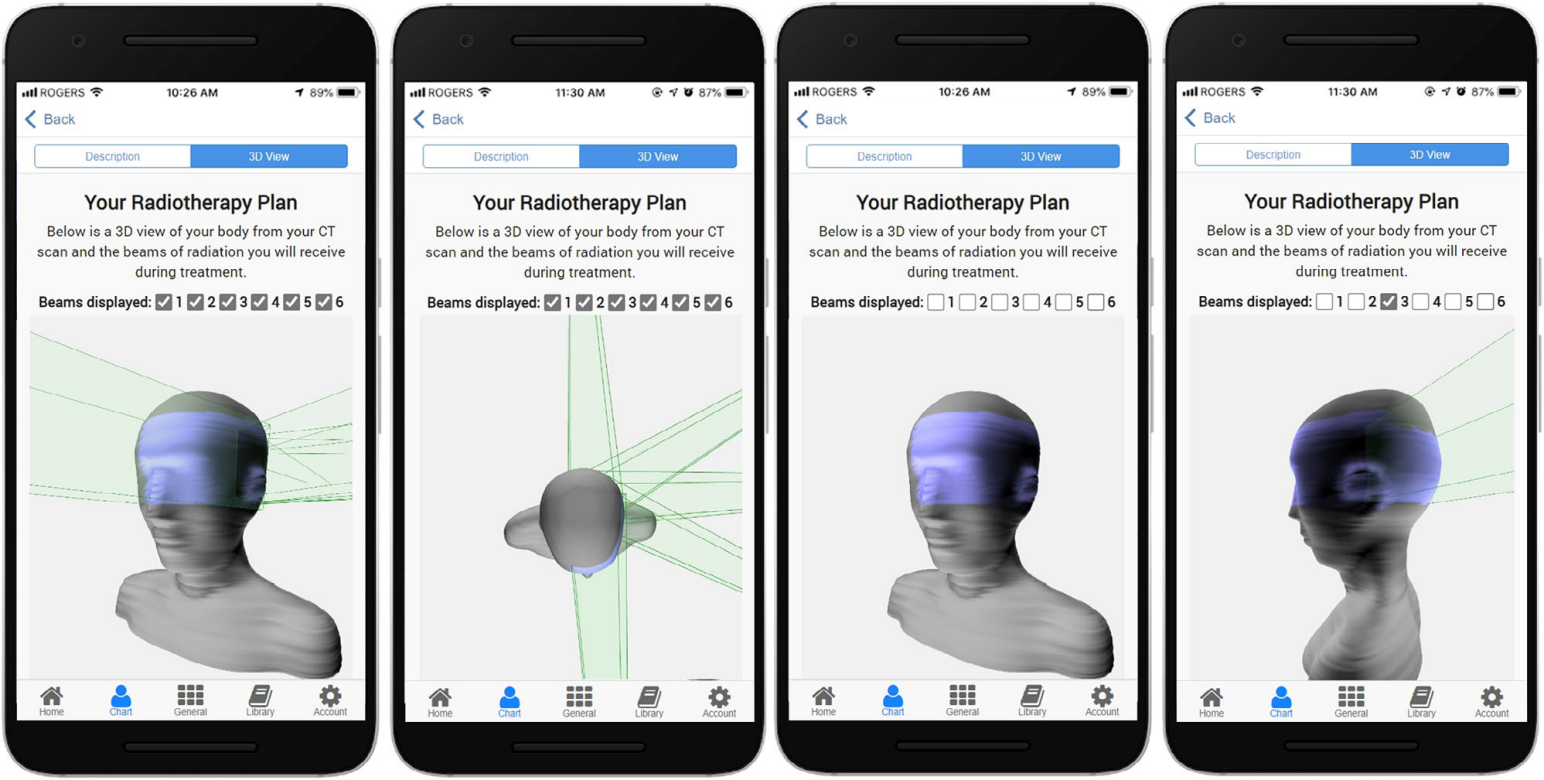
Screenshots of the 3D view of a head and neck cancer patient's radiotherapy treatment plan. This page can be opened by toggling to the “3D View” button at the top of the screen. Left to right, the screenshots show how the patient can change the viewing angle and zoom of their plan and toggle on and off its radiotherapy beams. The DICOM‐RT files used to generate this plan were retrieved from publicly available datasets in the SlicerRtData GitHub repository.^40^ ^.^

Figure 5 presents screenshots of the 3D view of a head and neck cancer patient's radiotherapy treatment plan. The screenshots demonstrate how the patient can use the interactive rotation and beam checkbox features on their plan.

#### 3.2.2 Post‐treatment summaries

To create the post‐treatment summary interface using mCODE data, sample patient data were sourced from Logica Health's FHIR Sandbox^42^ (Figure 3, inside the dashed red box). A set of extraction scripts were created to retrieve mCODE radiotherapy summaries from the sandbox via API calls, which are transferred as FHIR profiles in the JSON format. The scripts are then used to parse the mCODE data and insert them into the Opal Database. From there, the data are sent to the Opal App via the Listener where they are formatted into a structured text interface containing a treatment overview (treatment intent and reason, body site, etc.), followed by individual phase information for each phase in the overall treatment course (number of sessions, modality, technique, dates, and dose delivered to relevant volumes).

**FIGURE 6:**
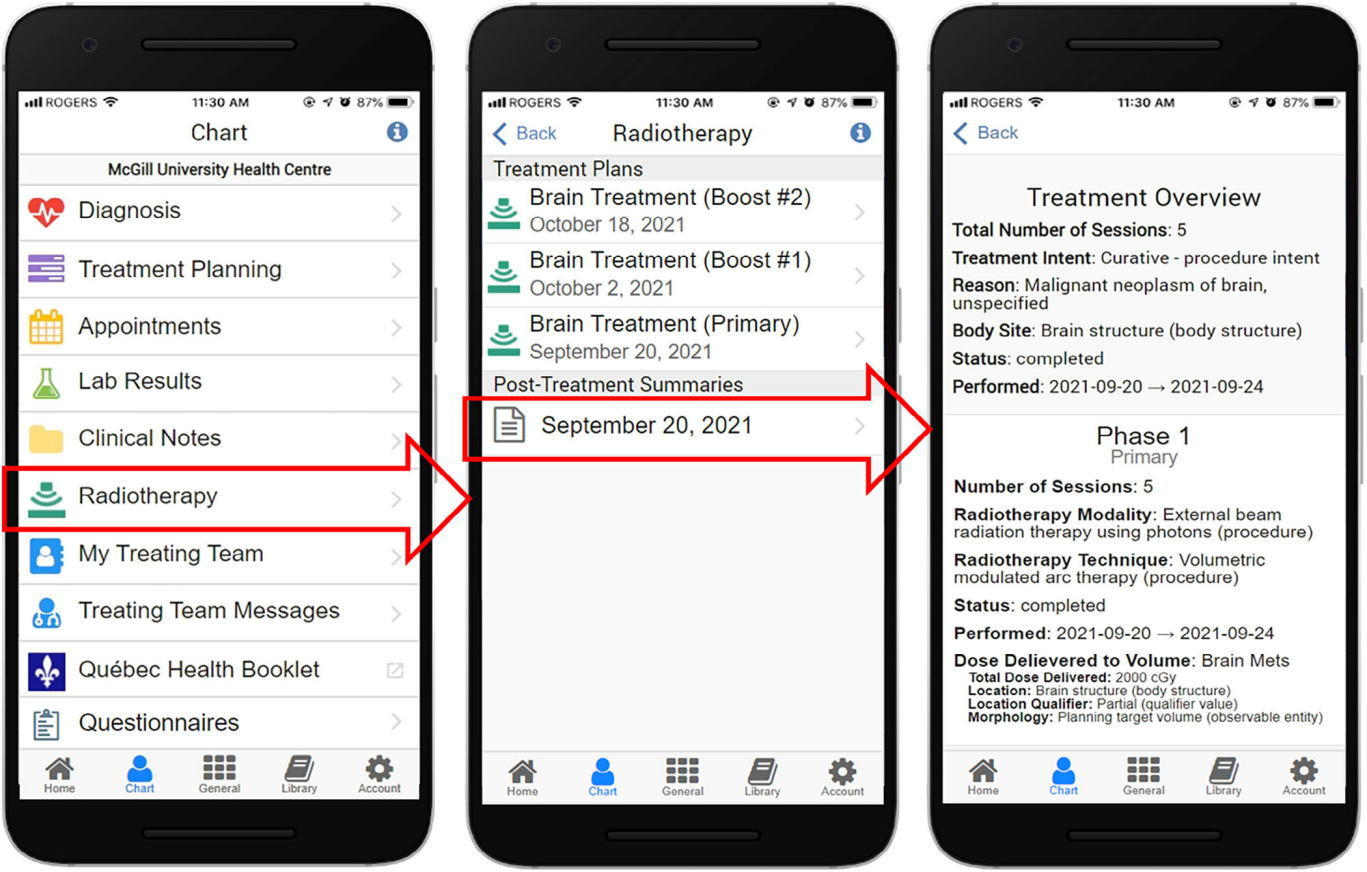
Sample of a post‐treatment radiotherapy summary generated from mCODE data. The radiotherapy menu is accessed via the Chart menu (leftmost screenshot) and the post‐treatment summaries are listed in the main radiotherapy menu (middle screenshot). Outlined red arrows show the interactive menu items that the user may tap on to open up the screenshots to their right. The data used to generate this treatment summary were retrieved from the Logica Health FHIR Sandbox.^42^ ^.^

Figure 6 provides screenshots of a sample post‐treatment summary generated and displayed in the Opal App using mCODE data.

### 3.3 Patient feedback

We received continuous feedback from our patient partners throughout the development process of this project. Consequently, they were highly satisfied with the finished prototype radiotherapy menu. They felt that the menu is intuitive to use and that its content would be both interesting and useful for radiotherapy patients generally. Our patient partners also highlighted that an important benefit of the menu is the ability to show their radiotherapy plans to family members and caregivers, including their family doctors.

Similarly, feedback gathered during our patient focus group was very positive. Participants were unanimously in agreement that they would have liked to have had this feature prior to their own radiotherapy treatments. Participants were asked if this feature would help them feel more prepared for their radiotherapy treatment and if it would impact their anxiety approaching their first treatment. Each answered enthusiastically, with responses such as “yes, definitely” and “[my anxiety] would have been decreased, for sure.” Participants felt that seeing their data in the radiotherapy menu would have better prepared them for their treatments because it “take[s] away a lot of the unknowns.”

Additionally, one participant noted that the radiotherapy menu would not only benefit radiotherapy patients prior to treatment, but also after treatment, as many patients would like to have a record of their completed treatment for future reference. In concluding the discussion on the radiotherapy menu, one patient stated, “I think people will love it!”.

## 4 DISCUSSION

In this study, we designed, developed, and implemented a novel prototype radiotherapy menu in a patient portal to improve patient understanding of and access to their radiotherapy data. Our radiotherapy menu received very positive feedback during a patient focus group. We attribute this positive reception to the participatory stakeholder co‐design approach we employed, which allowed patient partners to critique and approve each incremental design step, thereby resulting in a final prototype with high patient acceptability. To our knowledge, this is the first use of a patient portal to provide patients with access to and explanations of their personal radiotherapy treatment plans and the first use of the mCODE standard by a patient portal.

By using DICOM‐RT data, we were able to generate personalized treatment plan explanations and 3D visualizations of the patient's anatomy and planned treatment beams that can potentially be provided by any treatment planning system. Moreover, by leveraging the mCODE data standard, we were able to provide key radiotherapy summary data in a standardized and structured way that facilitates data exchange and interoperability.

Our patient partners and focus group participants unanimously felt that the radiotherapy menu would have decreased their radiotherapy‐related anxiety. Specifically, they felt that access to pre‐treatment plans can provide an intuitive explanation and interactive visualization of upcoming radiotherapy treatments that can help demystify the radiotherapy process, while the post‐treatment summaries have the potential to offer patients peace of mind knowing they would have a readily available record of their completed treatments. Our discussions with patients identified two specific components of the radiotherapy menu that contributed to this positive perception: (1) the manner in which the data are presented and (2) the ability for patients to access their data outside of the hospital on their own time.

In terms of the data presentation, we observed that patients were most appreciative of the interactive 3D renderings of their treatment beams. This is consistent with previous studies that have similarly reported on the benefits of showing patients 3D renderings of their radiotherapy treatment plans.^43^ For instance, Sulé‐Suso et al.^44^ and Wang et al.^45^ each conducted pilot studies using virtual reality to present patients with a 3D visual of their body and planned radiation beams. Each research group reported increased patient understanding and decreased radiotherapy‐related anxiety following the virtual reality consultations. However, one major drawback of this approach is that the sessions must be carried out at the hospital on special equipment with both the patient and staff present. This limitation not only requires additional resources, cost, and time from the hospital, but it is also not ideal for patients, as evidenced by Wang et al.,^45^ who reported that 70% of study participants desired to take home the data presented during the consultations.

In contrast, our radiotherapy menu requires only a smartphone and gives patients the flexibility to view their 3D plan wherever and as frequently as they like. Having their treatment plans accessible at all times allows patients to process the information at their own pace and to come to their first‐day treatment sessions feeling prepared and, as appropriate, with informed questions. Furthermore, it facilitates caregiver involvement, as patients can share and discuss their radiotherapy plans with care providers and family members outside of the hospital. Finally, patients can retain their post‐treatment summaries for future reference, either for their own interest or for medical purposes should they need to share them with other healthcare providers. Future work will explore functionality for patients to export their data in an interoperable way.

A notable limitation of this study is that our focus group consisted of only four participants. While our stakeholder co‐design approach of involving patient partners at all stages ensured we had a broadly patient‐acceptable solution, our findings may not be generalizable to all patients. Additionally, our process of recruiting focus group participants via the Opal patient portal itself may have introduced biases in our results since these patients were already accustomed to Opal and may be more inclined to actively participate in their care using it than Opal‐naive patients. In future work, we intend to conduct another focus group with a larger number of participants and include patients who do not currently use Opal. Nonetheless, our results demonstrate feasibility, and the patient feedback received, both during the stakeholder co‐design process and during the focus group, attest to this.

## 5 CONCLUSION

In conclusion, we successfully developed a novel prototype radiotherapy menu in a patient portal smartphone app to show patients their radiotherapy treatment plans and received very positive feedback on it during a patient focus group. In this study, we demonstrated that our solution meets patient education needs and is technically feasible. Our findings motivate future development on this project to integrate the prototype into the production version of the Opal patient portal.

## AUTHOR CONTRIBUTIONS

Conception and design: Kayla O'Sullivan‐Steben, Luc Galarneau, Susie Judd, Andrea M. Laizner_,_ Tristan Williams, John Kildea. Software development: Kayla O'Sullivan‐Steben. Analysis and interpretation: Kayla O'Sullivan‐Steben. Draft manuscript preparation: Kayla O'Sullivan‐Steben, John Kildea. All authors reviewed and approved the final version of the manuscript and agree to hold responsibility for the accuracy and integrity of the work.

## CONFLICT OF INTEREST STATEMENT

No conflicts of interest are reported. Opal is currently a purely academic endeavor but could potentially be commercialized in the future.
